# Supplementary material for: Variability in n-caprylate and n-caproate producing microbiomes in reactors with in-line product extraction
Source: mSystems. 2024 Jul 11;9(8):e00416-24. doi: 10.1128/msystems.00416-24 (PMC11334527; doi:10.1128/msystems.00416-24)
Supplement: Supporting Information — Supplemental figures and tables plus additional methodology. [file msystems.00416-24-s0003.pdf]

Supporting Information

Title: Variability in *n*-caprylate and *n*-caproate producing microbiomes in reactors with in-line product extraction.

Running Title: The microbiome of C8-producing reactors

Authors: Catherine M. Spirito[1,2*], Timo N. Lucas[3*], Sascha Patz[3], Byoung Seung Jeon[4], Jeffrey J. Werner[5], Lauren H. Trondsen[1], Juan J. Guzman[1], Daniel H. Huson[3], Largus T. Angenent[1,4,6,7,8]**

[1] Department of Biological and Environmental Engineering, Cornell University, Riley-Robb Hall, Ithaca, NY 14853 Ithaca, NY 14853, USA

[2] Office of Undergraduate Research, University of Maryland, College Park, MD, 20742, USA

[3] Institute for Bioinformatics and Medical Informatics, University of Tübingen, Sand 14, 72076 Tübingen, Germany

[4] Department of Geosciences, University of Tübingen, Schnarrenbergstr. 94-96, 72076 Tübingen, Germany

[5] Chemistry Department, SUNY-Cortland, Bowers Hall, Cortland, NY 13045, USA

[6] AG Angenent, Max Planck Institute for Biology Tübingen, Max Planck Ring 5, 72076 Tübingen, Germany

[7] Department of Biological and Chemical Engineering, Aarhus University, Gustav Wieds Vej 10D, 8000 Aarhus C, Denmark

[8] The Novo Nordisk Foundation CO2 Research Center (CORC), Aarhus University, Gustav Wieds Vej 10C, 8000 Aarhus C, Denmark

*Indicates shared first author status

**Correspondence to:

Largus T. Angenent
Email: l.angenent@uni-tuebingen.de

Number of pages: 14; Number of figures: 3; Number of tables: 3

Supporting Info - Methods

Gas Sparging Setup

Gas exit lines from the top of the reactor led to a condensation trap, bubbler, and then a gas flow meter (Calibrated Instruments Inc., Ritter MilliGas Counter Series MGC-1 V3.1, Hawthorne, NY). Stainless-steel lines were setup to sparge the reactor broth with either N_2, H_2, or both gases combined. The lines ran from tanks of N_2 or H_2 gas (pressure of tank set at 40 psi) to the reactor and entered the reactor near the reactor base. One-way check valves located near the reactor base were used to ensure uni-directional flow into the reactor. Gas flow meters (Cole-Parmer 65-mm Correlated Flowmeter, Part No. EX-03216-02), as well as needle valves, were used to control the flow rate of the gases into the reactor system. The gas flow rate entering the reactors was not measured. The gas flow rates leaving the reactors were measured and are reported in **Table 1** (in the main text).

Pertraction System

For product extraction, we used a setup similar to the one previously described by Agler et al. (1). Forward and backward membrane contactors (8.1 m^2 each, Membrana Liqui-Cel 4x13, X50 Membrane, Charlotte, NC, USA) were connected to the reactor setup (**Fig. S1**). A peristaltic pump (Cole Parmer 7553-30) was used to recirculate reactor broth at a flow rate of 48 mL min^{-1} through the shell-side of the forward contactor. Broth was removed from near the top of the reactor, passed through a filter (McMaster Carr High-Pressure Stainless Steel Y-Strainer, 1/2 NPT Female, 100 Mesh) to remove particulate matter, and then passed through the contactor and back to the reactor. The filter was periodically cleaned to remove the built-up particulate matter, on an approximately monthly basis. A mineral oil solvent with 30 g L^{-1} tri-*n*-octylphosphine oxide (TOPO) (Sigma Aldrich, St. Louis, MO, USA) was continuously circulated at a flow rate of ~30 mL min^{-1} (Cole Parmer 7553-30) through the lumen side of both the forward and backward membrane contactors. The purpose of the solvent was to primarily extract the more hydrophobic medium-chain carboxylates (as compared to the short-chain carboxylates) from the reactor broth. A well-mixed alkaline extraction solution was recycled at a flow rate of ~30 mL min^{-1} (Cole Parmer 7553-30) through the shell-side of the backward contactor. 0.3M sodium borate was initially used to buffer the extraction solution. The pH of the alkaline extraction solution was maintained at ~pH 9 *via* automated additions of 5 M NaOH using a pH controller and a corresponding base-addition pump.

Supporting Information – CSV Files

The following csv files are provided with the supporting info:

- RBOX gene and protein counts for all high-quality MAGs.
  - File name: rbox_mags.csv
- Bacterial microcompartments (BMCs) that were identified in reactor metagenomics samples using BMC caller.
  - File name: BMC_from_BMC_caller_tool.csv

Supporting Info - Tables

Table S1. Percent hydrogen (by volume) measured in the headspace of the three reactors. Mean and s.d. values are reported per main period.

	Period	H_2 (%)
Reactor 1	1	9.9 ± 5.2
	2	1.8 ± 0.6
	3	0.7 ± 0.2
Reactor 2	1	1.8 ± 1.9
	2	18 ± 19
	3	13 ± 5.3
Reactor 3	1	31 ± 9.6
	2	20 ± 14
	3	7.3 ± 4.6

Table S2. Effluent ethanol and carboxylate concentrations per period. Mean and standard error reported. Periods 1, 2, and 3 are abbreviated as P1, P2, and P3 in this Table.

		Ethanol (mM)	Acetate (mM)	*n*-Butyrate (mM)	*n*-Caproate (mM)	*n*-Caprylate (mM)
Reactor 1	P1	47.4 ± 3.9	3.97 ± 0.44	2.22 ± 0.11	2.26 ± 0.12	1.68 ± 0.12
	P2	61.0 ± 4.01	4.12 ± 0.39	5.26 ± 0.28	3.77 ± 0.17	0.98 ± 0.08
	P3	73.9 ± 4.12	7.92 ± 1.11	5.88 ± 0.54	3.75 ± 0.21	0.77 ± 0.07
Reactor 2	P1	29.1 ± 4.25	9.15 ± 0.90	4.20 ± 0.27	3.52 ± 0.22	0.97 ± 0.13
	P2	69.8 ± 7.54	11.7 ± 1.71	4.67 ± 0.34	3.68 ± 0.47	1.84 ± 0.34
	P3	115 ± 9.09	13.6 ± 1.32	2.33 ± 0.15	2.06 ± 0.12	0.54 ± 0.04
Reactor 3	P1	166 ± 9.69	2.55 ± 0.40	4.07 ± 0.53	1.58 ± 0.19	0.38 ± 0.04
	P2	119 ± 11.2	1.08 ± 0.18	2.39 ± 0.27	1.74 ± 0.17	0.48 ± 0.05
	P3	98.1 ± 5.86	3.40 ± 0.54	4.59 ± 0.47	4.05 ± 0.26	1.04 ± 0.07

Table S3: Components of stainless-steel reactor system. Vendor, catalog number, and a brief description provided.

Vendor	Catalog Number	Description	Purpose	Detailed purpose
McMaster Carr	52245K535	316 stainless steel compression tube fitting, adapter, for 3/8" tubing, 1/4" pipe	Connections	Effluent port
McMaster Carr	52245K824	Stainless steel compression tube fitting adapter for 3/8 NPT, 3/8" tube OD	Connections	System recycle
McMaster Carr	52245K825	Stainless steel compression tube fitting adapter for 1/2 NPT, 3/8" tube OD	Connections	Internal recycle
Swagelok	MS-PTS-6	Swak Pipe Thread Sealant, 6 cc	Connections	Sealing
MMC	7098K25	Slim Spade-Terminal Relay SPDT, 12 DC Control Voltage	Electrical	Liquid level
McMaster Carr	7527K53	Terminal block	Electrical	Electrical connections (on plywood)
McMaster Carr	7098K23	Socket with spring clamps (for relay)	Electrical	Liquid level
McMaster Carr	2779K5	LED Panel mounting indicating light	Electrical	liquid level
ebay		Power strips	Electrical	Electrical connections (on plywood)
ebay		12 V DC power supply	Electrical	Liquid level
MMC	7527K59	Jumpers (pack of 25)	Electrical	Liquid level
MMC	7527K836	Cover for terminal block	Electrical	Electrical connections (on plywood)
MMC	70355K85	Power Cord with Three-Blade Plug 18 Gauge Wire, 6' Long SPT-1 Cord	Electrical	Control board
MMC	7196K41	Straight-Blade Three-Blade Female Connector, NEMA 5-15	Electrical	Control board
MMC	8054T15	Stranded Wire 300V AC, 18 Gauge, 200 ft, Black	Electrical	Control board
MMC	8054T15	Stranded Wire 300V AC, 18 Gauge, 200 ft, Red	Electrical	Control board
MMC	8054T15	Stranded Wire 300V AC, 18 Gauge, 200 ft, White	Electrical	Control board
Quantumflow Tech		4x13 liqui-cel membrane contactors	Extraction	Extraction

McMaster Carr	51525K236	Plastic Quick-Turn (Luer Lock) Coupling, Polypropylene, Female x Male Thread, 1/4"-28 UNF Thread (Pack of 10)	Filter	Filter
McMaster Carr	44205K21	Disposable Water Filter (5 microns)	Filter	Filter
Lowes	253209	Toilet Flange Extender and Complete Spacer (2 spacers)	Filter	Strainer
Lowes	23493	4-in PVC Cap Fitting	Filter	Strainer
Lowes	126605	4 1/2 in dia Stainless steel basket	Filter	Strainer
Lowes	137639	1/4" Bolts	Filter	Strainer
Lowes	67340	1/4" Nuts	Filter	Strainer
Lowes	61814	1/4" Washers	Filter	Strainer
MMC	5670K85	Type 303 Stainless Steel Barbed Tube Fitting, 1/4 NPT, 3/8" ID tube	Filter	Strainer
MMC	4452K672	Locknut 1/4 NPT	Filter	Strainer
MMC	91525A145	Large diameter flat washer for 1/2" screw size, pack of 10	Filter	Strainer
Airgas	HY HP300	H_2 gas tank	Gas	
Ritter US LLC		Series MGC-1 Milli Gas Counter V 3.1	Gas	Gas measurement
McMaster Carr	89785K43	316 stainless steel tubing 3/8" OD	Lines	Liquid and gas lines
McMaster Carr	89785K122	316 stainless steel tubing, 1/4" OD	Lines	pH lines?
McMaster Carr	4066K41	Stainless steel pressure gauge (0-30 psi)	Lines	Pressure measurements
Omega	LV-11	Liquid level switch, stainless steel	Liquid level	Liquid level
Omega	PHE-7353-15	pH probe	pH	Probe
Cole Parmer	YO-56705-00	pH controller	pH	Controller
Cole Parmer	07554-80	Pump drive	Pumps	
Cole Parmer	77250-62	High pressure pump head	Pumps	
Cole Parmer	S-95564-24	High pressure tubing	Pumps	
Digikey	AE9869-ND	CABLE DB9M-DB9M 2M (for pumps)	Pumps	For back connection

McMaster Carr	44635K644	316 stainless steel unthreaded pipe size 4, 6' length	Reactor	Reactor body
McMaster Carr	44695K39	316 stainless steel low pressure unthreaded flange	Reactor	Reactor body
McMaster Carr	44695K119	316 stainless steel cap	Reactor	Not needed
McMaster Carr	45555K121	Reducing coupling; pipe size 4 to 1 1/2"	Reactor	Bottom
McMaster Carr	9472K49	All-purpose gasket	Reactor	
McMaster Carr	91236A804	Znc-Pltd STL Low-Strength Hex Head Cap Screw 5/8"-11 Thread, 2-1/2" Length, packs of 10	Reactor	Reactor flange
McMaster Carr	4452K112	Stainless steel coupling, 1/4 NPT	Reactor	Gas outlet, effluent, extra port
McMaster Carr	4452K138	Stainless steel half coupling, 1/8 NPT	Reactor	Temp
McMaster Carr	4452K114	Stainless steel coupling for 1/2 pipe size	Reactor	
McMaster Carr	52245K539	Stainless steel compression tube fitting adapter for 1/2 NPT, 1/2" tube OD	Reactor	Not needed
McMaster Carr	90108A035	Washers for 5/8" screw size, pack of 25	Reactor	Reactor flange
McMaster Carr	95462A533	Nuts for 5/8" screw size, pack of 50	Reactor	Reactor flange
McMaster Carr	4942K7	Plastic sight glass (window diameter 2 13/16"), for 3" tube OD	Reactor	Window
McMaster Carr	50485K165	Adapter, quick clamp to tube	Reactor	Window
McMaster Carr	4322K155	Clamp	Reactor	Window
McMaster Carr	43315K27	PTFE gasket for reactor window	Reactor	Window
McMaster Carr	4452K213	Half coupling 3/4" pipe size	Reactor	pH port

McMaster Carr	4452K112	Coupling for 1/4 pipe size	Reactor	Effluent, extra port
McMaster Carr	4452K139	Half coupling 1/4" pipe size	Reactor	Gas line
McMaster Carr	4452K111	Coupling for 1/8 pipe size	Reactor	Temp
McMaster Carr	4942K3	Plastic sight (reactor window) for tube OD 2"	Reactor	Window
McMaster Carr	43315K25	PTFE gasket for reactor window (2")	Reactor	Window
McMaster Carr	50485K163	quick clamp tube fittings for tube OD 2"	Reactor	Window
McMaster Carr	4322K153	Clamp (2")	Reactor	Window
McMaster Carr	47865K41	Brass ball valve 1/4 NPT, female x male	Reactor	Not needed?
McMaster Carr	7768K22	Brass check valve (female inlet x male outlet, 1/4 pipe size)	Reactor	
McMaster-Carr	4322K714	Clamp for reactor window (bolted)	Reactor	
McMaster Carr	44635K832	316 stainless steel unthreaded pipe size 4, 3' length	Reactor	Body
McMaster Carr	45555K122	Reducing coupling; pipe size 4 to 2"	Reactor	
McMaster Carr	50485K521	Stainless Steel Quick-Clamp Tube Fitting, Female Pipe Adapter for 1" Tube OD, 1" NPT Pipe	Reactor	
McMaster Carr	4322K152	Stainless Steel Quick-Clamp Tube Fitting, Wing-Nut Clamp for 1 &1-1/2" Tube OD, 1.984" Flange	Reactor	
McMaster Carr	4509K13	Buna-N Gasket for Sanitary Tube Fitting for 1" Tube OD	Reactor	
McMaster	4452K125	Stainless steel square head plug for 3/4 pipe size	Reactor	Temporary
McMaster Carr	4452K113	Stainless steel coupling, 3/8 NPT	Reactor	System recycle out
McMaster Carr	4452K138	Half stainless steel coupling, 1/8 NPT	Reactor	For liquid level
McMaster Carr	4452K421	45 deg. stainless steel elbow, female, 1/8 NPT	Reactor	For liquid level

McMaster Carr	4548K111	Stainless steel thread nipple, 1/8 NPT (close)	Reactor	For liquid level
MMC	4452K111	Stainless steel 1/8 NPT coupling	Reactor	For liquid level
MMC	4816K153	Stainless steel pipe (1/8 NPT, threaded both ends, 20" length)	Reactor	For liquid level
MMC	4816K121	Stainless steel pipe (1/8 NPT, threaded both ends, 18" length)	Reactor	For liquid level
MMC	4548K115	Stainless steel pipe (1/8 NPT, threaded both ends, 3" length)	Reactor	For liquid level
Home Depot	ERZ782478W-4	Commercial shelving unit; 77 in W x 78 in H x 24 in D for reactor stand	Stand	
Lowes		Paint	Stand	
Lowes		Primer	Stand	
Home Depot		Plywood	Stand	
McMaster-Carr	98750A214	5/8" threaded rod for reactor stand/setup	Stand	
McMaster Carr	90108A035	Washers for 5/8" screw size, pack of 25	Stand	
McMaster Carr	95462A533	Nuts for 5/8" screw size, pack of 50	Stand	
McMaster Carr	90322A335	5/8" thread steel rod for reactor stand (6 ft)	Stand	
MMC	35765K277	Heat sheet (3"x12")	Temp	Heater
Omega	HSTC-TT-K-20S-120	Type K Hermetically sealed thermocouple, 3 m (120"), stripped leads	Temp	For under heater
MMC	5556K39	Rigid high temperature fiberglass pipe insulation	Temp	Insulation
MMC	45325K114	Plastic jacketing for pipe insulation	Temp	Insulation
MMC	29695T123	Cinching strap	Temp	Insulation
ebay		Heat transfer paste, 1 oz	Temp	Heater
Amazon		Dual Digital Display PID Temperature Controller SSR(2 Alarms)	Temp	Temp control
Omega	TJ36-CASS-18U-6	Thermocouple, type K, stainless steel, 6 " length, 1/8" diameter	Temp	For reactor
Omega	SSLK-18-18	1/8 x 1/8 compression fitting	Temp	For reactor temp

MMC	3869K35	Thermocouple and RTD Connector Male, 400 Deg F, Flat-Pin, Type K, Yellow	Temp	Heater
MMC	3869K34	Thermocouple and RTD Connector Female, 400 Deg F, Flat-Pin, Type K, Yellow	Temp	Heater

Supporting Info - Figures

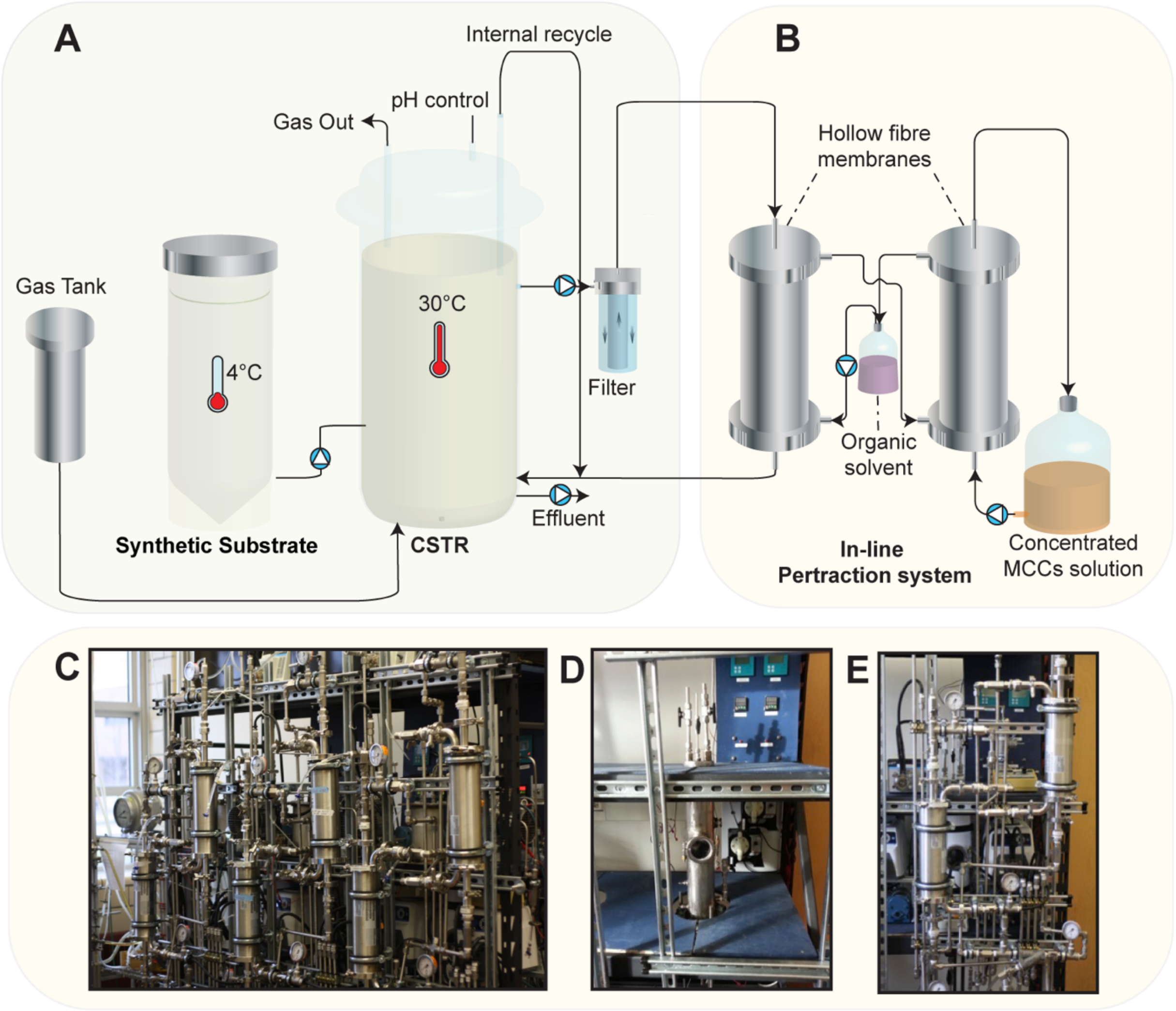

Figure S1. Diagram showing setup of each reactor. Gas was sparged into the bottom of the reactor during Periods 2 and 3 (**A**). Pertraction system setup with membrane contactors, oil, and alkaline extraction solution (**B**). Photos of the setup of the in-house constructed, three stainless-steel reactors: the membrane contactors and stainless-steel lines of the pertraction system are shown (**C**); stainless steel CSTR and electrical control board (**D**) and single reactor with pertraction system (**E**).

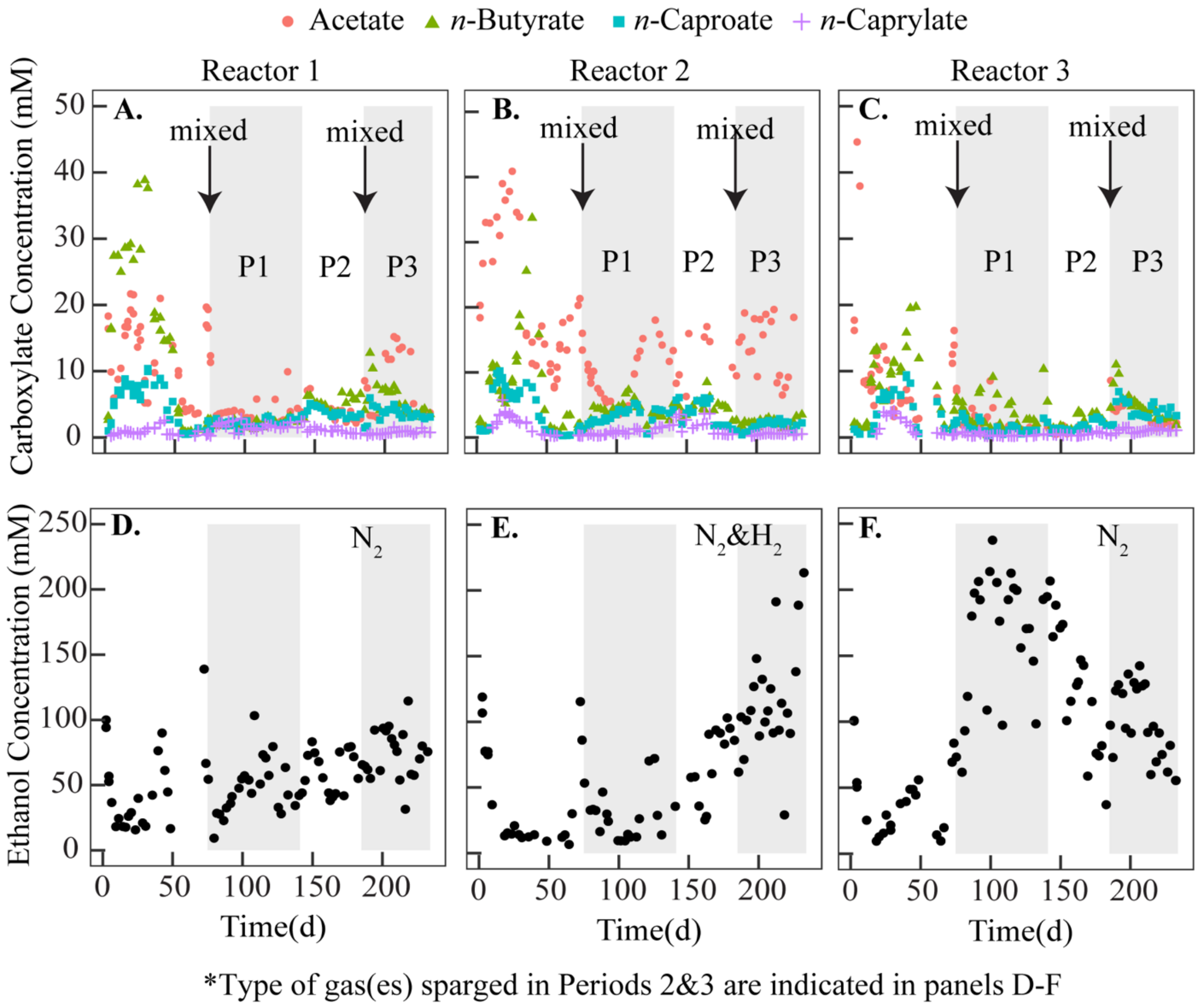

Figure S2. Effluent carboxylate concentrations throughout the operating period for Reactor 1 (**A**), Reactor 2 (**B**), and Reactor 3 (**C**). The two-time points at the start of Periods 1 and 3, when the biomass from all the reactors was mixed, are indicated on these panels. Effluent ethanol concentrations throughout the operating period for Reactor 1 (**D**), Reactor 2 (**E**), and Reactor 3 (**F**) are also shown. The type of gases that were sparged into these reactors during Periods 2 &3 are indicated in these panels. Dark shading in panels **A-F** indicate Periods 1, 2, and 3 (P1, P2, and P3), which are the main periods reported on in this text.

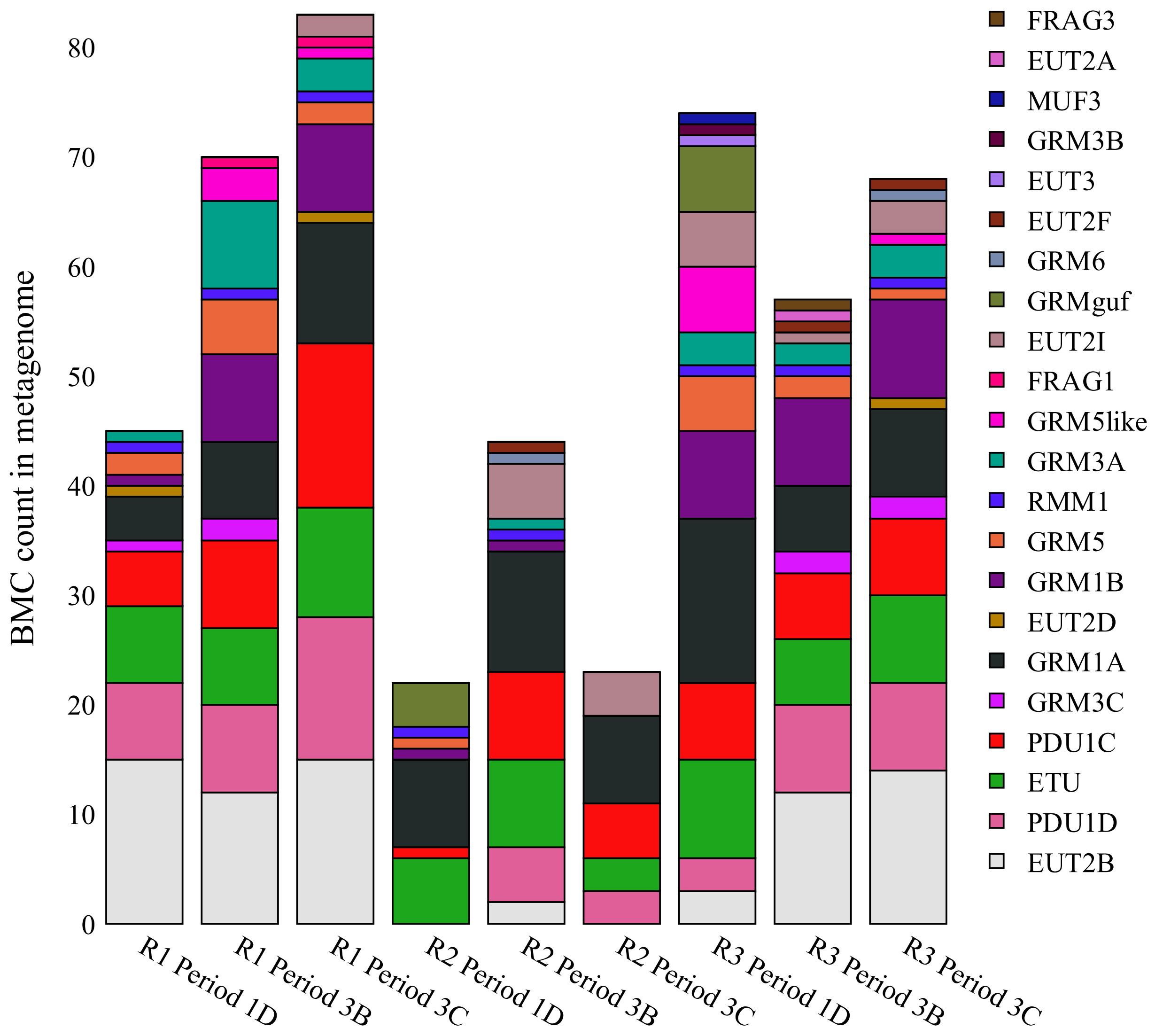

Figure S3. Count of bacterial microcompartment (BMC) shell proteins in annotated metagenomes. Each sampling point is represented by a distinct bar and the bar heights are proportional to the number of microcompartments found. The bar segments are colored by the microcompartment's type. R1-3 are Reactors 1-3. The periods are explain in the methods of the main text.

References

1. Agler MT, Spirito CM, Usack JG, Werner JJ, Angenent LT. 2012. Chain elongation with reactor microbiomes: upgrading dilute ethanol to medium-chain carboxylates. Energy & Environmental Science 5:8189-8192.
